# Changing risk of spring frost damage in grapevines due to climate change? A case study in the Swiss Rhone Valley

**DOI:** 10.1007/s00484-018-1501-y

**Published:** 2018-01-24

**Authors:** Michael Meier, Jürg Fuhrer, Annelie Holzkämper

**Affiliations:** 1 Division Agroecology and Environment, Agroscope, CH8046, Zurich, Switzerland; 2 Oeschger Center for Climate Change Research, University of Bern, Bern, Switzerland

**Keywords:** Climate change, Frost risk, Grapevine, Swiss Rhone Valley, Phenology

## Abstract

Late spring frost is a severe risk during early plant development. It may cause important economic damage to grapevine production. In a warming climate, late frost risk either could decline due to the reduction in frost days and an advancement of the last day of frost or increase due to a more pronounced shift forward of the start of the active growing period of the plants. These possibilities were analyzed in a case study for two locations in the lower Swiss Rhone Valley (Sion, Aigle) where viticulture is an important part of agriculture. Twelve phenology models were calibrated for the developmental stage BBCH09 (bud burst) using measured or reconstructed temperature data for two vineyards in Changins (1958 to 2012) and Leytron (1977 to 2014) together with observed phenological data. The day of year (DOY) for BBCH09 was then modelled for the years 1951 to 2050 using the best performing phenology model in combination with ten downscaled and bias-corrected climate scenarios. A 100-day period starting with BBCH09 was defined, during which daily mean and minimum temperatures were used to calculate three frost risk indices in each year. These indices were compared between the periods 1961–1990 (reference) and 2021–2050 (climate change scenario). Based on the average of the ensemble of climate model chains, BBCH09 advanced by 9 (range 7–11) (Aigle) and 7 (range 5–8) (Sion) days between the two time periods, similar to the shift in the last day of frost. The separate results of the different model chains suggest that, in the near future, late spring frost risk may increase or decrease, depending on location and climate change projections. While for the reference, the risk is larger at the warmer site (Sion) compared to that at the cooler site (Aigle), for the period 2021–2050, small shifts in both phenology and occurrence of frost (i.e., days with daily minimum temperature below 0 °C) lead to a small decrease in frost risk at the warmer but an increase at the cooler site. However, considerable uncertainties remain that are mostly related to climate model chains. Consequently, shifts in frost risk remain uncertain for the time period considered and the two study locations.

**Electronic supplementary material:**

The online version of this article (10.1007/s00484-018-1501-y) contains supplementary material, which is available to authorized users.

## Introduction

Late spring frost is a severe risk during early plant development. Although these events do not happen very often, they are an important threat, for instance, to wine production in the top wine regions of Europe, e.g., in France, Spain, and Italy. In Switzerland, where wine production contributed CHF 575 (5.1%) and 633 (5.6%) million to the total agricultural revenue in the years 2015 and 2016, respectively (Swiss Federal Statistical Office 2016), the most recent frost events causing damage to grapevines occurred in the Swiss Rhone Valley in spring 2012 (Favre and Balleys 2012) and more widespread during April 2017, after an exceptionally warm period in March. In the Swiss Rhone Valley, as one of the main production regions, seven frost events were recorded since 1950, all causing severe damage to grapevines. In this inner alpine valley, 48.8 km^2^ is used for wine production (2015) with a revenue of CHF 150 million. The main variety for white wine is ‘Chasselas,’ being cultivated on 9.1 km^2^ of the total 18.8 km^2^ used for the production of white grape varieties (Office de la Viticulture 2016).

For frost damage to occur, the timing of early phenological plant development in relation to the occurrence of low-temperature events is crucial. After spring de-acclimation, most sensitive stages of grapevine development are around budburst. As both climatological frost conditions and grapevine phenology are related to the seasonal development of temperature, they are subject to long-term changing climatic conditions. Thus, in the future, the risk for frost damage depends not only mainly on the change in the frequency and occurrence of frost days but also on the shifting phenology of grapevines. Rigby and Porporato (2008) showed that increasing daily temperatures (i.e., a positive temperature trend) lead to a decrease in spring frost risk, while an increasing temperature variance increases the risk. However, according to Webb et al. (2012), the same climate change projection may have different effects on plant phenology in different regions. In their study in Australia, they found earlier ripening for grapevines in nine of ten regions, while ripening in one region was delayed. Thus, the future risk of frost damage to grapevines depends on the magnitude of the change in regional climate, production region, and variety.

Many studies have shown that the phenology of many plant species advances due to increasing temperatures (Menzel et al. 2006). With regard to grapevines, this advancement has been observed or modelled, for instance, in Germany (Bock et al. 2011), Luxembourg (Urhausen et al. 2011), France (Jones and Davis 2000), and Australia (Webb et al. 2007, 2012). This trend is expected to continue with rising temperatures, while at the same time, the number of frost days declines (IPCC 2013). However, these trends may not lead to a reduction in spring frost risk (Ball et al. 2012). An earlier start of the growing period could also lead to an increased risk of frost damage in situations where late spring frost days advance at a slower rate than the rate of the advancement of phenology (Fuhrer et al. 2014; Mosedale et al. 2015). But it remains uncertain, which factor will dominate with regard to grapevines cultivated at different locations.

Projections for future frost risk may also depend on the choice of the index used to quantify the risk. Different indices exist for grapevines, which are either based on observations, models, or experiments (Favre and Balleys 2012; Ferguson et al. 2013; Fuller and Telli 1999; Molitor and Junk 2013; Molitor et al. 2014; Snyder and Melo-Abreu 2005; Mosedale et al. 2015). Temperature thresholds for frost damage not only vary between studies but also depend on the phenological phase and grapevine variety. For the phase of budburst (BBCH09, according to Hack et al. 1992; Lorenz et al. 1994), Snyder and Melo-Abreu (2005) consider 30 min of − 3.9 and − 8.9 °C as being lethal to 10 and 90%, respectively, for the relatively frost-resistant variety ‘Concorde.’ Ferguson et al. (2013) reported a temperature of − 1.2 °C as being lethal to 50% of the plant parts in varieties such as Sauvignon Blanc, Chardonnay, Pinot Gris, and Gewürztraminer. The experiments conducted by Fuller and Telli (1999) identified temperatures between − 3 and − 3.5 °C as being lethal to about 60% of the plants, regardless of variety. Molitor and Junk (2013) and Molitor et al. (2014) used the thresholds of 0 °C in their work on frost risk in the Mosel wine regions in Germany and Luxembourg, and Mosedale et al. (2015) considered thresholds of 0 and 2 °C.

The aim of the present study was to assess future changes in frost risks in grapevine based on ten regional climate change projections for the lower Swiss Rhone Valley derived from GCM–RCM chains of the EU FP7 ENSEMBLES project, all of them assuming the SRES A1B emission scenario (Gobiet et al. 2014) as applied in the Intergovernmental Panel on Climate Change Fourth Assessment Report (IPCC AR4). While recent research focuses on RCP scenarios in combination with the more complex climate models, as used in IPCC AR5, Knutti and Sedláček (2013) have shown that projected temperature changes as computed by the new models are remarkably similar to the projections our study is based on. Additionally, we account for climate model uncertainty by applying ten different GCM-RCM chains. Regarding the climate change projections, we specifically focused on the two major wine-growing areas around Aigle and Sion with slightly different current climates, and on the variety ‘Chasselas’ as this region’s main white vine variety. We developed a phenology model to simulate budburst and used the model to quantify the shift in the timing of BBCH09 between the reference period 1961–1990 and a future period between 2021 and 2050. Using the same climate information, changes in three different frost indices were calculated and related to the corresponding timing of budburst.

## Methods and data

### Study locations

Located in the southwest of Switzerland, the Swiss Rhone Valley lies in the Western Alps and covers an area of 5225 km^2^. Two sites were selected for the present analysis.

- Aigle (AIG) located at 6.9 E/46.3 N at an altitude of 382 m above sea level (asl), with an annual mean temperature of 9.8 °C and an average number of frost days per year of 77.7 (1981–2010) (www.meteoswiss.ch), and
- Sion (SIO) located at 7.3 E/46.2 N at 481 m asl, with an annual mean temperature of 10.1 °C and an average number of frost days per year of 93.6 (1981–2010) (www.meteoswiss.ch)

### Meteorological data

Phenological observations to develop and validate the phenological model were not available for AIG and SIO, but instead for several other locations. For two vineyards in Changins (CGI) and Leytron (LEY), temperature was reconstructed to fit the timespan of observed phenological data from 1958 to 2012 for CGI and 1977 to 2014 for LEY. Data from the Agrometeo Service (www.agrometeo.ch) were used for CGI for the years 1998 to 2015 and for LEY for the years 2003 to 2014, and Meteoswiss (www.meteoswiss.ch) data for the stations Pully (PUY), Geneva (GVE), Evionnaz (EVI), and Sion (SIO) for the periods 1978 to 2015, 1957 to 2015, 1993 to 2014, and 1976 to 2015, respectively (Table 1).

**Table 1:** Overview of the meteorological stations providing temperature data (Changins (CGI), Pully (PUY), Geneva (GVE), Leytron (LEY), Evionnaz (EVI), and Sion (SIO)), and their distance to the corresponding study location

Station	CGI	PUY	GVE	LEY	EVI	SIO
Organization	Agrometeo	MeteoSwiss	MeteoSwiss	Agrometeo	MeteoSwiss	MeteoSwiss
Distance	0 km	36 km	19 km	0 km	15 km	9 km
Period	1998–2015	1978–2015	1957–2015	2003–2014	1993–2014	1976–2014
Altitude	440 m asl	455 m asl	420 m asl	512 m asl	481 m asl	482 m asl
Coordinates	46° 24′ N/6° 14′ E	46° 31′ N/6° 40′ E	46° 15′ N/6° 08′ E	46° 11′ N/7° 13′ E	46° 11′ N/7° 02′ E	46° 13′ N/7° 20′ E
Data use	Input for phenology models for CGI	Temperature reconstruction for CGI	Temperature reconstruction for CGI	Input for phenology models for LEY	Temperature reconstruction for LEY	Temperature reconstruction for LEY

Source: Agrometeo (www.agrometeo.ch/) and MeteoSwiss (www.meteoswiss.admin.ch)

Since the phenological observations covered the years 1958 to 2012 for CGI and 1977 to 2014 for LEY, but temperature recordings only started in 1998 and 2003, respectively, daily mean temperatures from 1957 to 2015 for CGI were calculated based on two linear regressions: The first one, Eq. 1, covers the timespan from 1978 to 1997 and uses data from the Meteoswiss stations in Pully (PUY) and Geneva (GVE). The second regression, Eq. 2, only uses the MeteoSwiss data from GVE as input variables to reconstruct temperatures from 1957 to 1977. The years 1998 to 2015 were covered by the Agrometeo recordings in CGI.

1 $$ \widehat{\mathrm{CGI}}={b}_{0,\mathrm{bivar}}+{b}_{1,\mathrm{bivar}}\times GVE+{b}_{2,\mathrm{bivar}}\times \mathrm{r}.\mathrm{PUY} $$

With,

$$ \widehat{\mathrm{CGI}} $$
Estimated mean temperature for CGI
*b*_0, bivar_: Intercept of bivariate linear regression for $\widehat{\mathrm{CGI}}$
*b*_1, bivar_: Slope of bivariate linear regression for $\widehat{\mathrm{CGI}}$
*b*_2, bivar_: Slope of bivariate linear regression for $\widehat{\mathrm{CGI}}$
GVE: Observed mean temperature for GVE
r. PUY: Residual mean temperature for PUY

2 $$ \widehat{\mathbf{CGI}}={\boldsymbol{b}}_{\mathbf{0}}+{\boldsymbol{b}}_{\mathbf{1}}\times \mathbf{GVE} $$

With,

$$ \widehat{\mathrm{CGI}} $$
Estimated mean temperature for CGI
*b*_0_: Intercept of linear regression for $\widehat{\mathrm{CGI}}$
*b*_1_: Slope of linear regression for $\widehat{\mathrm{CGI}}$
GVE: Observed mean temperature for GVE

Regarding LEY, two linear regressions were calculated to obtain daily mean temperatures for the years 1976 to 2014: To cover the timespan from 1993 to 2002, data from the MeteoSwiss stations in Evionnaz (EVI) and Sion (SIO) were used in a first regression, Eq. 3. Thereafter, MeteoSwiss data from SIO served as input to reconstruct temperatures from 1976 to 1992 with a second regression, Eq. 4. Again, the data for the remaining years 2003 to 2014 was provided by the Agrometeo recordings for LEY.

3 $$ \widehat{\mathbf{LEY}}={\boldsymbol{b}}_{\mathbf{0},\mathbf{bivar}}+{\boldsymbol{b}}_{\mathbf{1},\mathbf{bivar}}\times \mathbf{SIO}+{\boldsymbol{b}}_{\mathbf{2},\mathbf{bivar}}\times \mathbf{r}.\mathbf{EVI} $$

With,

$$ \widehat{\mathrm{LEY}} $$
Estimated mean temperature for LEY
*b*_0, bivar_: Intercept of bivariate linear regression for $\widehat{\mathrm{LEY}}$
*b*_1, bivar_: Slope of bivariate linear regression for $\widehat{\mathrm{LEY}}$
*b*_2, bivar_: Slope of bivariate linear regression for $\widehat{\mathrm{LEY}}$
SIO: Observed mean temperature for SIO
r. EVI: Residual mean temperature for EVI

4 $$ \widehat{\mathbf{LEY}}={\boldsymbol{b}}_{\mathbf{0}}+{\boldsymbol{b}}_{\mathbf{1}}\times \mathbf{SIO} $$

With,

$$ \widehat{\mathrm{LEY}} $$
Estimated mean temperature for LEY
*b*_0_: Intercept of linear regression for $\widehat{\mathrm{LEY}}$
*b*_1_: Slope of linear regression for $\widehat{\mathrm{LEY}}$
SIO: Observed mean temperature for SIO

For both bivariate regressions (i.e., reconstructing temperature in CGI with PUY and GVE, as well as data from EVI and SIO for LEY), the two input variables were highly correlated (*ρ*(PUY, GVE) and *ρ*(EVI, SIO)). Therefore, one of each pair of variables was partialled out and the resulting residuals (i.e., r.PUY and r.EVI) were used instead, Eqs. 5 and 6, respectively. This was done again by applying two linear regressions followed by the calculation of the residual for each observation, Eq. 7 (PUY) and Eq. 8 (EVI), respectively.

5 $$ \mathbf{r}.\mathbf{PUY}=\mathbf{PUY}-\widehat{\mathbf{PUY}} $$

With,

r. PUY: Residual mean temperature for PUY
PUY: Observed mean temperature for PUY
$$ \widehat{\mathrm{PUY}} $$
Estimated mean temperature for PUY

6 $$ \mathbf{r}.\mathbf{EVI}=\mathbf{EVI}-\widehat{\mathbf{EVI}} $$

With,

r. EVI: Residual mean temperature for EVI
EVI: Observed mean temperature for EVI
$$ \widehat{\mathrm{EVI}} $$
Estimated mean temperature for EVI

7 $$ \widehat{\mathbf{PUY}}={\boldsymbol{b}}_{\mathbf{0},\mathbf{part}.\mathbf{out}}+{\boldsymbol{b}}_{\mathbf{1},\mathbf{part}.\mathbf{out}}\times \mathbf{GVE} $$

With,

$$ \widehat{\mathrm{PUY}} $$
Estimated mean temperature for PUY
*b*_0, part. out_: Intercept of linear regression for partialling out *ρ*(PUY, GVE)
*b*_1, part. out_: Slope of linear regression for partialling out *ρ*(PUY, GVE)
GVE: Observed mean temperature for GVE

8 $$ \widehat{\mathbf{EVI}}={\boldsymbol{b}}_{\mathbf{0},\mathbf{part}.\mathbf{out}}+{\boldsymbol{b}}_{\mathbf{1},\mathbf{part}.\mathbf{out}}\times \mathbf{SIO} $$

With,

$$ \widehat{\mathrm{EVI}} $$
Estimated mean temperature for PUY
*b*_0, part. out_: Intercept of linear regression for partialling out *ρ*(EVI, SIO)
*b*_1, part. out_: Slope of linear regression for partialling out *ρ*(EVI, SIO)
SIO: Observed mean temperature for GVE

For the four regressions to obtain estimates for CGI and LEY, the datasets for CGI and LEY were each split into a calibration and a validation set, consisting of 60 and 40% of the whole dataset, respectively. The partialling out was done by computing a linear regression on the whole samples of PUY and EVI, since a validation was not required. To evaluate the regressions, the statistics *R*^2^ and adjusted *R*^2^ were used. Additionally, the RMSE was calculated for the validating data set for all four regressions (CGI ~ GVE + PUY, CGI ~ GVE, LEY ~ SIO + EVI, and LEY ~ SIO).

### Phenological data

Phenological data was obtained through Agroscope Viticulture Research Centre Pully (https://www.agroscope.admin.ch/). The focus was on the timing of budburst and thus on the phenological phase BBCH09 (Bloesch and Viret 2008). All observations came from two Swiss vineyards (Table 2) located in CGI near Lake Geneva and LEY in the Swiss Rhone Valley. Observations for the variety ‘Chasselas’ from CGI covered the period from 1958 to 2012, and those from LEY the period from 1977 to 2014. This resulted in sample sizes of 55 and 38 years, respectively, covering 88 observations of budburst in total.

**Table 2:** Overview of the vineyards from where the phenological data was derived. The locations of the vineyard in Changins (CGI) and Leytron (LEY) are defined in terms of their coordinates, altitude, and region. Additionally, the number of observations regarding budburst (BBCH09) and the corresponding period, as well as average yearly rainfall and global radiation, is provided

	CGI	LEY
Region	Lake Geneva Region	Swiss Rhone Valley
Coordinates	46° 24′ N/6° 14′ E	46° 11′ N/7° 13′ E
Altitude	440 m asl	512 m asl
Mean annual rainfall (2009–2015)	993 mm	595 mm
Mean annual global radiation (2009–2015)	1186 kW m^−2^	1392 kW m^−2^
Observation period	1958–2012	1977–2014
Number of observations for BBCH09	55	38

Source: Agroscope’s Viticulture Research Centre Pully (www.agroscope.admin.ch/); Agrometeo (www.agrometeo.ch)

### Phenological model

According to Chuine (2000) and Fila et al. (2014), two main model types are widely used to describe grapevine phenology: Forcing (F) models and chilling-and-forcing (CF) models. In F models, forcing units (FU) with respect to temperature are summed up starting at the same day every year according to a certain date or length of day (i.e., photoperiod). By fixing the start to a specific day of year (DOY), F models propose that breaking of dormancy solely depends on photoperiod and temperatures. In CF models, the starting point regarding FU accumulation is allowed to change from year to year, depending on a threshold for the accumulation of chilling units (CU). Consequently, CF models depend on winter temperature and can react to mild winters by starting FU accumulation at a later DOY, which implies that breaking of dormancy depends on both cold and warm temperatures.

Photoperiod (i.e., length of daylight period) can influence plant phenological development (Fennell and Hoover 1991; Ferguson et al. 2011, 2013; Vitasse and Basler 2013). Here, we applied the first two model types and clustered them according to Basler (2016) into forcing models excluding photoperiod (F models), F models including photoperiod (F.PP models), chilling-and-forcing models excluding photoperiod (CF models), and chilling-and-forcing models including photoperiod (CF.PP models) (Table 3). Due to the limited sample size (i.e., 88 observations), only models using six parameters or less were used. This allowed separating the sample into a calibration and a validation part of 63 (i.e., 71.6%) and 25 (i.e., 28.4%) observations, respectively. The photoperiod was calculated according to Fischer et al. (2014). All models tested are described in the “Supplementary Material.”

**Table 3:** Applied clusters of phenology models. Models starting accumulation of forcing units (FU) at a specific day of year (DOY) are forcing (F) or photoperiod incorporating forcing (F.PP) models. Models starting FU accumulation after reaching a threshold for chilling units (CU) are chilling-and-forcing (CF) or chilling-and-forcing and photoperiod incorporating (CF.PP) models

	Starting DOY according to length of day	Starting DOY according to summed CU
Temperature	F models	CF models
Temperature and photoperiod	F.PP models	CF.PP models

Two widely used functions in F models are the growing degree-day function (GDD) according Murray et al. (1989) and the sigmoid function of mean temperature (SF), as described by Hänninen (1990) and Kramer (1994). Fila et al. (2014) obtained good results with phenology models when calculating forcing with the beta-type function (Yan and Hunt 1999; Amaducci et al. 2008) (see “Supplementary Material”).

Calibration was done with R (Version 3.1.0; R Development Core Team, 2014), using the method of simulated annealing (Bélisle 1992) and by minimizing the squared error (SE). Since sample size was always the same for all models and fitting of parameters, minimizing standard error (SE) had the same effect as minimizing mean square error (MSE) or root mean square error (RMSE). After calibration, the different models were run with the identical validation sample to compare the goodness of fit between models. This was done by calculating the RMSE, the unbiased RMSE (RMSE′), and the Nash-Sutcliffe Efficiency Index (NSE). The best performing model was used for the following frost risk analysis.

### Indices for frost risk

Risk of spring frost damage was described by three indices. All indices were calculated from daily minimum (*T*_min_) or daily mean temperature (*T*_mean_) during a 100-day period starting from the DOY of BBCH09.

- Index 1: DOY with daily *T*_min_ below 0 °C (*T*_min_ < 0) (last day of frost, LDF)
- Index 2: DOY of the last with daily *T*_mean_ below 2 °C (*T*_mean_ < 2)
- Index 3: sum of daily temperature deltas below the threshold of 2 °C (Σ(*T*_mean_ < 2 °C)

### Climate model chains

Projections for *T*_min_ and *T*_mean_ were derived from simulations with ten climate model chains according to model ensembles runs conducted for the European Union Framework 6 ENSEMBLES project (http://www.ensembles-eu.org/) (Table 4). Each model chain consisted of a regional climate model (RCM), driven by a global climate model (GCM), based on the emission scenario A1B (Christensen et al. 2010; Nakicenovic and Swart 2000). The model chains were calibrated with the E-OBS gridded observational dataset with daily data for a 25 × 25 km grid (Haylock et al. 2008; Klein Tank et al. 2009). Using the quantile mapping approach (Themeßl et al. 2010), the calibrated datasets were downscaled and error corrected for different climate stations in the Alpine region within the framework of the EU ACQWA project (http://www.acqwa.ch) (Haylock et al. 2008; Smith et al. 2014; Wilcke et al. 2013). Covering a timespan from 1951 to 2050, the years 1961 to 2000 served as a calibration and validation period, leaving the years 2001 to 2050 as modelled future data (Mendlik et al. 2011). Data for the periods 1961–1990 (reference) and 2021–2050 (CC scenario) were extracted and used in the analysis.

**Table 4:** Climate model chains

	Acronym	RCM	GCM
1	DMI_HIRHAM_ECHAM5	HIRHAM	ECHAM5
2	ICTP_RegCM_ECHAM5-r3	RegCM	ECHAM5
3	KNMI_RACMO_ECHAM5-r3	RACMO	ECHAM5
4	MPI_REMO_ECHAM5-r3	REMO	ECHAM5
5	SMHI_RCA_ECHAM5-r3	RCA	ECHAM5
6	METNO_HIRHAM_HadCM3Q0	HIRHAM	HadCM3Q0
7	ETHZ_CLM_HadCM3Q0	CLM	HadCM3Q0
8	HC_HadRM3Q0_HadCM3Q0	HadRM3Q0	HadCM3Q0
9	SMHI_RCA_HadCM3Q3	RCA	HadCM3Q3
10	UCLM_PROMES_HadCM3Q0	PROMES	HadCM3Q0

Source: Christensen et al. (2010); http://ensemblesrt3.dmi.dk/extended_table.html (accessed on 27 October 2016)

## Results

### Phenology models

**Fig. 1:**
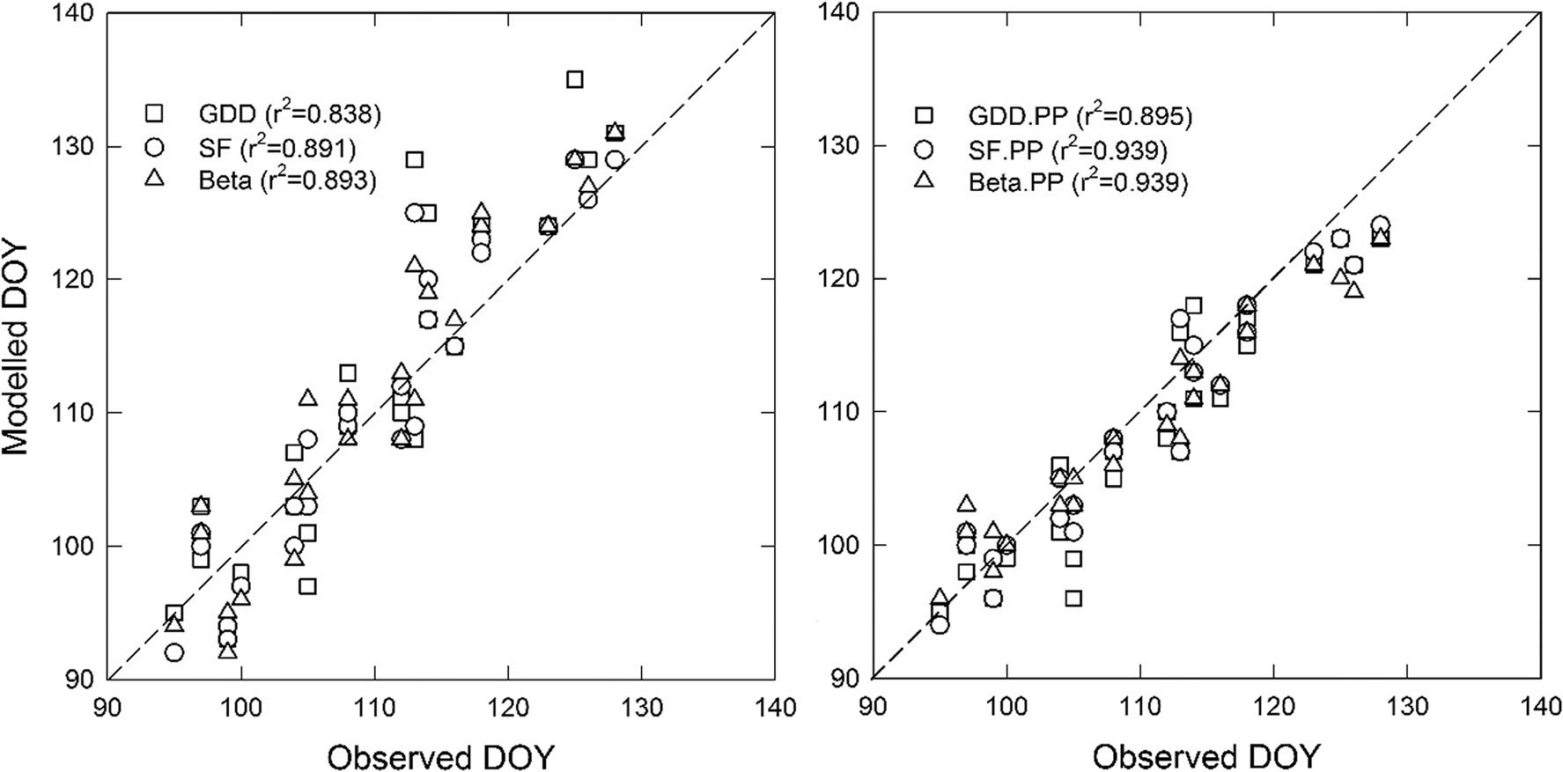
Relationship between observed and modelled day of year (DOY) for budburst (BBCH09) using three forcing models (F, left panel) and three photoperiod incorporating forcing (F.PP, right panel), using either growing degree-day (GDD), sigmoid (SF), or beta-type (Beta) functions for forcing units. The dashed lines represent the 1:1 relationship

Validation of the calibrated phenology models resulted in RMSE/RMSE′ values of 3 to 6 days and NSE values of 0.92 to 0.62. Models incorporating photoperiod clearly outperformed forcing models that excluded photoperiod. As an example, Fig. 1 shows the relationship between observed and modelled DOY for BBCH09 for three F and three F.PP models. The performance of chilling-and-forcing models excluding photoperiod (i.e., CF models) and chilling-and-forcing models including photoperiod (i.e., CF.PP models) was lower (not shown). The ranking of the first six models was similar for all statistics used. In further analyses, the SF.PP model was used.

### DOY for budburst

**Fig. 2:**
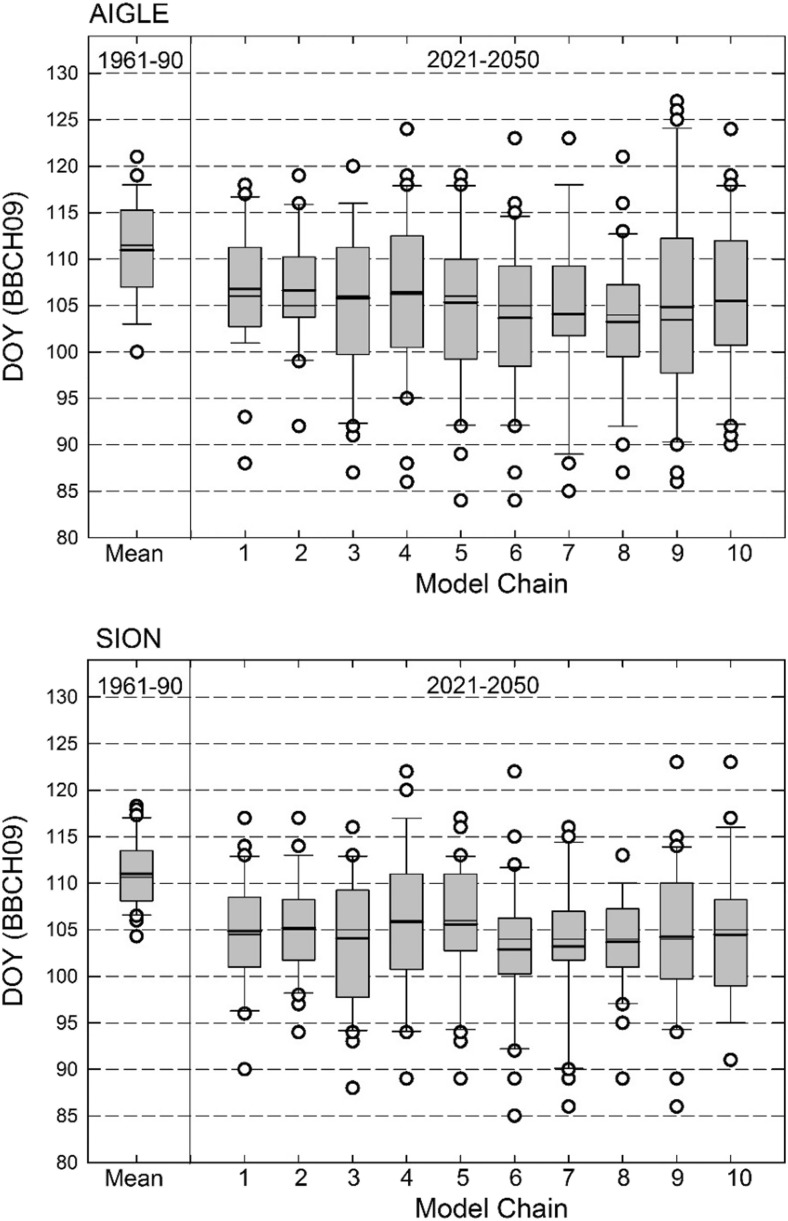
Simulated DOY for BBCH09 for the two locations for the 30-year reference and the CC scenario periods using all model chains. Boxes show mean (thick line), median (thin line), and 25th/75th percentiles, whiskers are 10th/90th percentiles, and outliers are marked as circles. Number of model chains according to Table 2

The SF.PP model was used to calculate DOY of BBCH09 for the two locations for the reference and the CC scenario period. While all model chains projected an advancement of the DOY relative to the mean of the reference period, the plot in Fig. 2 illustrates the small spread in the mean between model chains for the CC scenario period, but considerable difference in the variance. For the reference period, the mean DOY was 114 for AIG and 111 for the slightly warmer site SIO. The average of all model projections was 105 for AIG and 104 for SIO. On average, DOY for BBCH09 advanced by 9 (range 7–11) (AIG) and 7 (range 5–8) (SIO) days between the two time periods.

### Indices 1 and 2 vs BBCH09

**Fig. 3:**
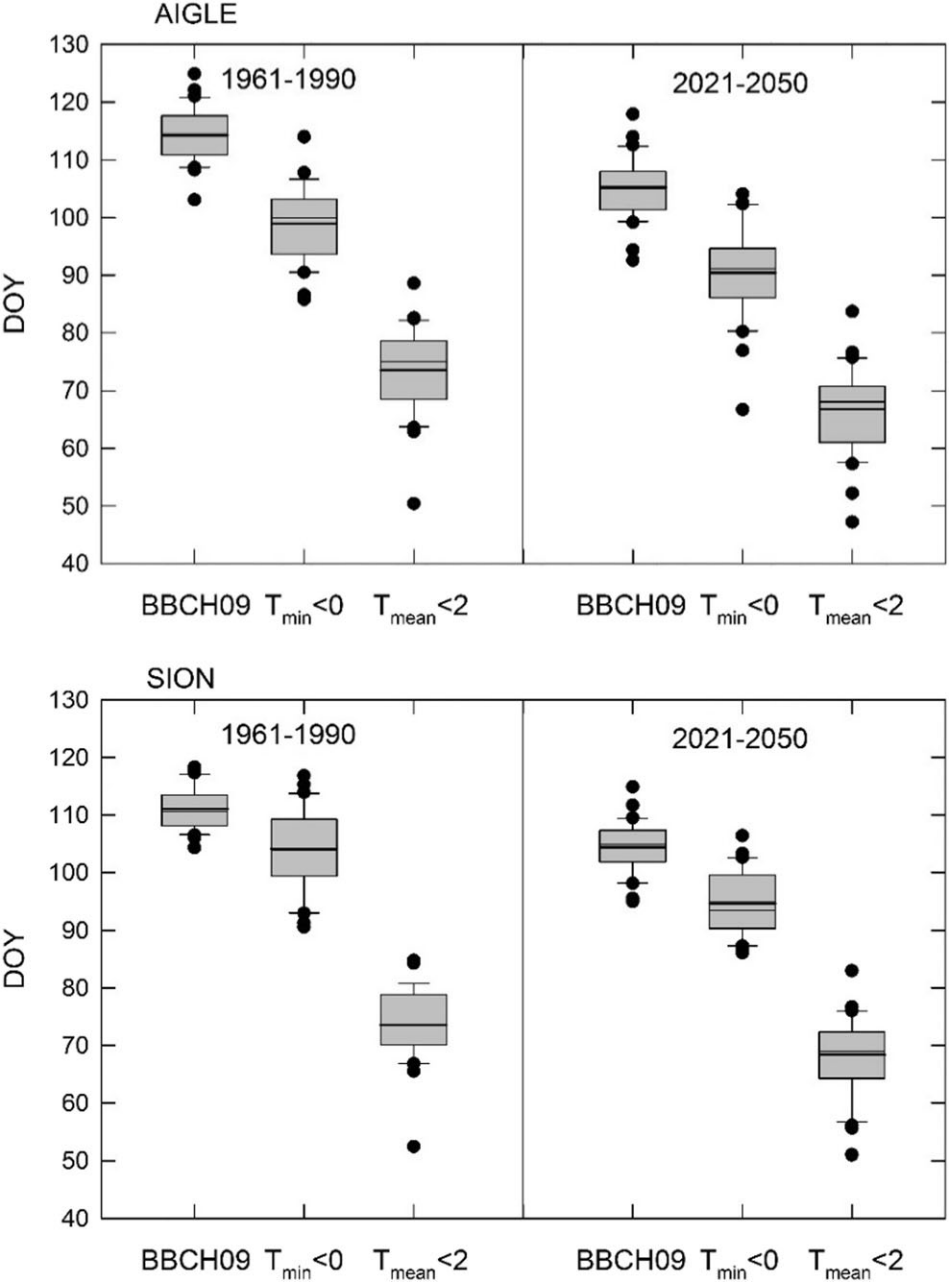
Comparison between the DOY for BBCH09 and DOY for Index 1 and Index 2 for the reference and the CC scenario periods using 30-year data from the model ensemble for both locations. Boxes show mean (thick line), median (thin line), and 25th/75th percentiles, whiskers are 10th/90th percentiles, and outliers are marked as circles

The model ensemble mean for BBCH09 was compared with that of Index 1 and Index 2 to check the overlap between shifts in the critical phenological stage and in frost risk indicators. Relative to the reference period and for both locations, the advancement in BBCH09 was paralleled by an advancement in the means of both indices (Fig. 3). However, an overlap of the distributions was only evident for Index 1, while frost according to Index 2 would always occur later than BBCH09. The overlap for Index 1 was larger for SIO than for AIG. The mean shift in Index 1 from the reference to the CC scenario period was from DOY 99 to 90 (AIG) and from DOY 103 to 95 (SIO), and was thus in same range as the shift in BBCH09 (see Fig. 2). The mean difference between DOY for BBCH09 and for Index 1 for the reference and the CC scenario period was 12 and 15 days (AIG) and 8 and 10 days (SIO), respectively. The coefficient of variation for the difference increased between reference and CC scenario from 0.24 to 0.43 at AIG and decreased from 0.77 to 0.36 at SIO.

### Model intercomparison for Index 1 vs BBCH09

**Fig. 4:**
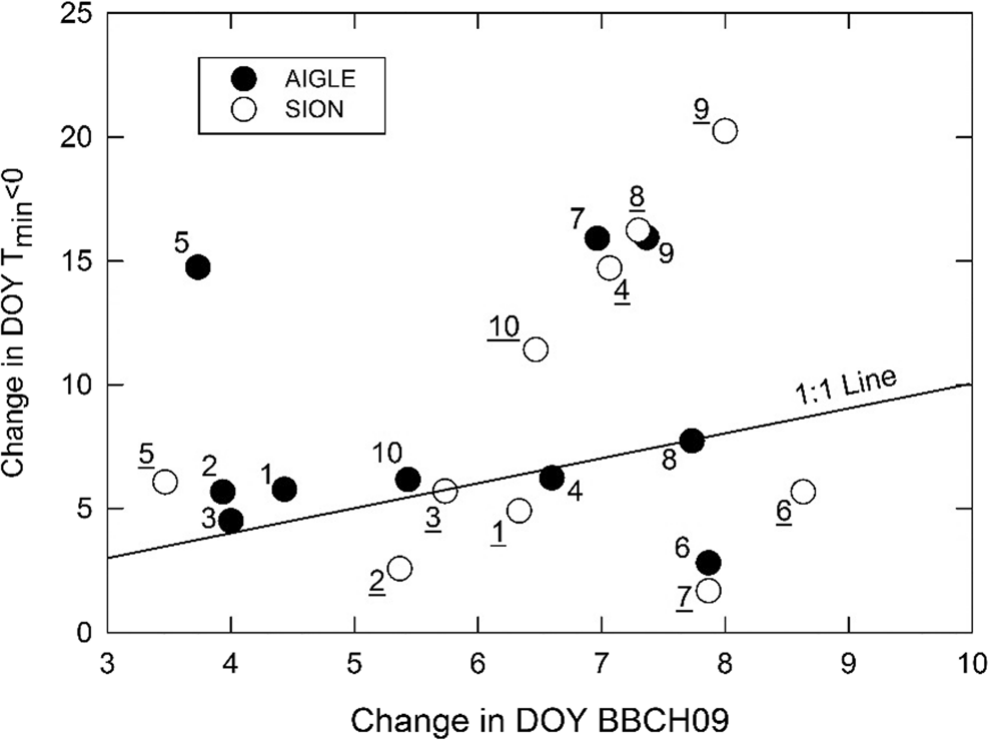
Relationship between the mean change in DOY for BBCH09 and for Index 1 (*T*_min_ < 0) for each model chain. Numbers refer to model chains according to Table 1 (underlined numbers for SIO, not underlined numbers for AIG)

The difference in the timing between DOY for BBCH09 and for Index 1 was subject to the uncertainty associated with model chains (Fig. 4). While the majority of model chains confirmed similar shifts for both BBCH09 and Index 1 (i.e., points located close to the 1:1 line), there are notable exceptions in both directions. Four model chains for SIO and three for AIG projected much stronger shifts in Index 1 than in DOY for BBCH09, thus indicating decreased risk of frost damage, while another set of model chains projected the opposite, particularly for SIO. For SIO, the model chain projecting the largest decrease in risk was SMHI_RCA_HadCM3Q3 (9), and the model chain projecting the largest increase in risk was ETHZ_CLM_HadCM3Q0 (7).

### Probability of frost risk

**Fig. 5:**
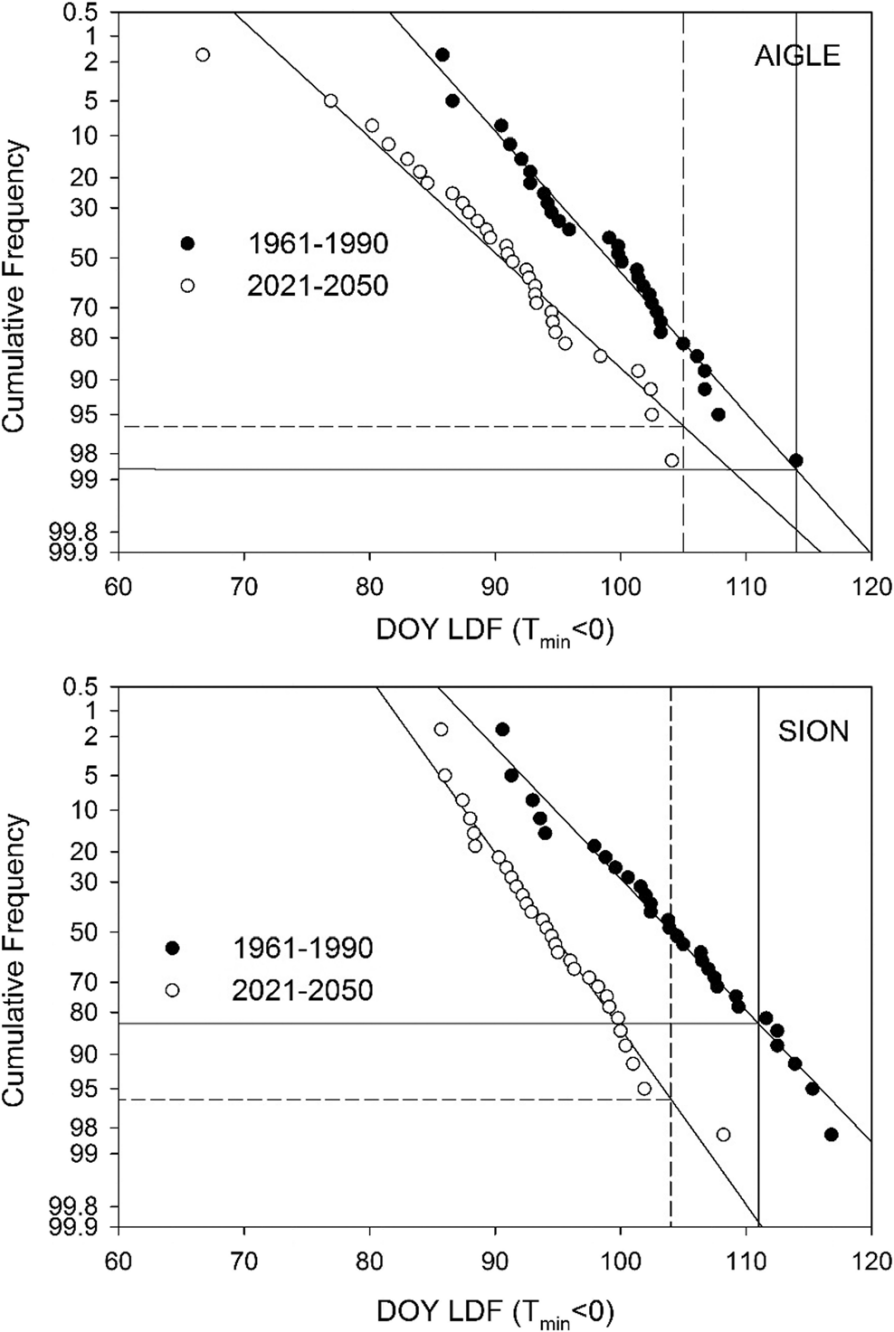
Normalized probability plots for DOY of the last frost day (LDF, Index 1) for AIG and SIO using means of model ensemble data for the reference and the CC scenario period. Vertical lines indicate the DOY for BBCH09 and horizontal lines the associated probability of occurrence of the last frost day (LDF) as reflected by Index 1 (*T*_min_ < 0)

The risk of frost damage using Index 1 was further investigated by using normalized frequency distribution plots (Fig. 5). This analysis confirmed that for the reference period the probability of frost to occur after budburst was projected to be larger at SIO than that at AIG. However, the plot also shows that for SIO the risk was smaller for the CC scenario period compared to the reference. At AIG, the opposite was the case, although with a very small difference in probability between the two time periods.

**Fig. 6:**
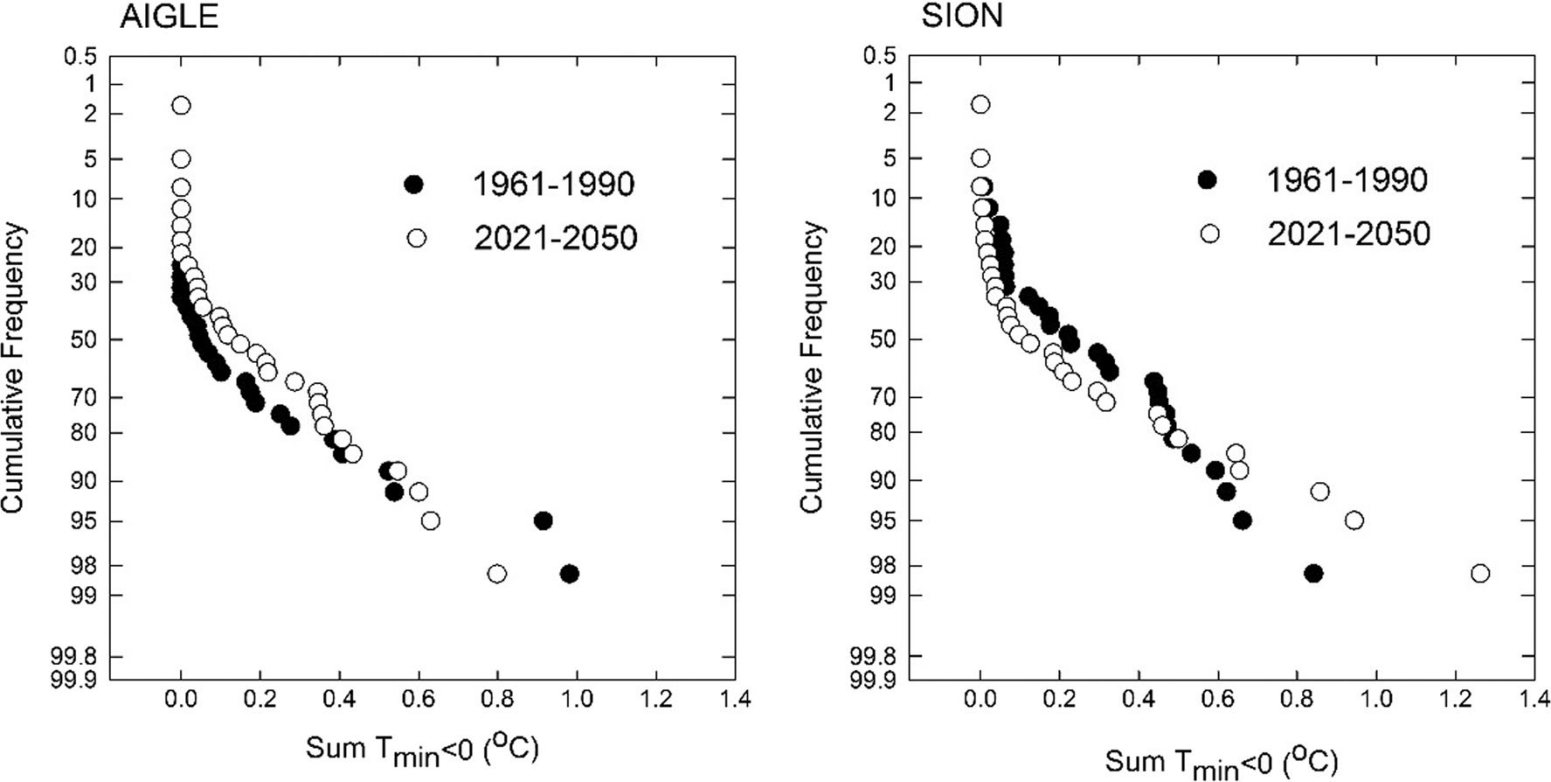
Normalized probability plots for the sum of mean temperatures below 2 °C (Index 3) for AIG and SIO using means of model ensemble data for the reference and the CC scenario period

A similar analysis was done for Index 3, which combines both probability and hazard by adding up daily temperature deltas below a threshold of 2 °C (Fig. 6). The plot for the ensemble mean for AIG and SIO indicates only minor shifts in the probability distribution between reference and CC scenario, but some differences between sites. At AIG, a trend towards lower frequencies appeared for the CC scenario for the lower values of the Index, and the reverse was the case for the higher values. The opposite appears for SIO. This indicates that for the reference period the stronger frost events are more likely at SIO than at AIG, but could become more frequent at AIG and less frequent at SIO.

## Discussion

The results for the two selected locations in an important wine production region of Switzerland confirm the generally projected advancement of the timing of budburst with climate change. The ensemble mean shift in DOY for BBCH09 between the reference and the CC scenario period is 7 to 9 days. This shift seems reasonable, although it is at the lower end of the range proposed by Fila et al. (2014) who projected BBCH09 to advance between 14 and 44 days during the period 1990 to 2090. However, our projections only cover the time until 2050. Most likely, the shift in the DOY for BBCH09 would continue towards earlier dates, if the applied temperature projection would cover a longer time period. However, the climate model projection uncertainty would also increase substantially for further time horizons. It is assumed here that for frost damage to occur, critical temperatures are required during a 100-day period after budburst. For the 30-year reference period, the probability for frost damage based on Index 1 is around 17% for SIO and only 2% for AIG (Fig. 5). For the years 1950–2015, six events of frost damage in the Swiss Rhone Valley are documented, which occurred in the years 1957, 1974, 1991, 1995, 1997, and 2012 (Favre and Balleys 2012), which is broadly in agreement with the probability calculated for SIO. For the CC scenario, the last frost day (Index 1) advances by 8 and 9 days between the two periods, and is thus similar to the shift in BBCH09 for the two locations. The difference between the time of the last day of frost and BBCH09 is much larger for AIG than for SIO, both for the reference and the CC scenario periods, thus indicating a larger risk for frost to occur at SIO. This is in agreement with the larger number of frost days at this site during the 1981–2010 period (www.meteoswiss.ch). The variability (i.e., coefficient of variation) regarding the mean difference between DOY for BBCH09 and DOY of the last frost day, however, increases at AIG and decreases at SIO. Rigby and Porporato (2008) observed an increase in variability with climate change and concluded that the risk for spring frost damage would increase. Hence, our result suggests a higher but decreasing risk of frost damage with climate change at SIO and a lower but increasing risk at AIG. This conclusion is supported by the probability analysis in Fig. 6.

The observed advancement of both BBCH09 and last day of frost (Index 1) is in line with that in the studies by Molitor et al. (2014) and Molitor and Junk (2013) for the Mosel wine region in Luxembourg and Germany, respectively. However, calibrated for the grapevine variety ‘Müller-Thurgau’ and based on the SRES A1B scenario, Molitor et al. (2014) modelled the DOY for BBCH09 to advance by 11 days between the period 1961 to 1990 and 2059 to 2098, but the DOY of the last day of frost (*T*_min_ < 0 °C) after BBCH09 to advance more significantly by 28 days, thus indicating a stronger decrease in frost damage risks as observed here for SIO. They calculated the frequency of frost days during a 60-day period starting with BBCH09 to decrease from, on average, 0.137 to 0.082 events per year between the period 1961 to 1990 and the period 2021 to 2050. Similar studies exploring frost risk changes for other fruit trees produced results that are broadly in line with the findings from this study. For example, Di Lena et al. (2017) suggest that the risk of spring frost damage for almonds grown in Central Italy is likely to remain unchanged in the future. Eccel et al. (2009) suggest a constant or reduced risk of spring frost damage for apples in Northern Italy. Also, Hoffmann and Rath (2013) project that blossom frost risk is unlikely to increase until the end of the century for apple trees in the northern regions of Germany. A recent study by Vitasse et al. (2018) confirmed that spring frost risk to fruit and forest trees has remained unchanged at lower elevations in Switzerland, while it has significantly increased at higher elevations over the last few decades—likely due to an insignificant change in the occurrence of the latest spring frost event at higher elevations. A slow decrease and unchanged spring frost risk was also identified for cherries grown at two sites in Germany (Chmielewski et al. 2017). Our projections and those of Molitor et al. (2014) are not in line with those of the study published by Mosedale et al. (2015) who projected for southwest England the DOY for BBCH09 to advance by 45 days by the year 2080 under the SRES A1B scenario. This large shift led to an increase in the probability for a frost day (*T*_min_ < 0) after BBCH09 from 17% for the years 2010 to 2039 to 24% for the years 2040 to 2069. The discrepancy between results from studies at different locations underlines that the change in frost risk depends on location, but may also be affected by the choice of the phenology model, specifically consideration of effects of photoperiod on the potential phenological advancement under climate change.

There are several uncertainties associated with the results of our simulations. These concern mainly (a) the temperature reconstruction, (b) the phenology model, and (c) the CC model chains. The applied method to reconstruct daily mean temperature at CGI and LEY is straight forward: mean temperatures from one, respectively two other meteorological station serve as independent variables, after correction for their correlation (i.e. partialling out). However, the method is much less sophisticated than the method applied by Frei (2014), which considers the non-linear profile of the vertical temperature curve as well as non-Euclidean distances, considering hills and valleys. Still, the goodness of fit according to RMSE is reasonable. While here the RMSE ranges from 0.5 to 0.8 °C, in the applied cross-validation for the entire Swiss Alpine Region, Frei (2014) computed RMSE between 0.9 and 2.3 °C.

Sophisticated phenology models contain many parameters, thus sufficiently large calibration and validation samples are needed. For this reason, models with more than six parameters were not included here. Additionally, the phenology models in this study only considered daily mean temperature and some of them, daily photoperiod. The length of the photoperiod may limit the advancement of phenological development in response to increasing temperature, which is not accounted for by using only temperature in modelling the start of the potential growing season, as done in Fuhrer et al. (2014) or Molitor et al. (2014). There are additional factors controlling phenology such as water availability, CO_2_ concentration, and nutrients (Martínez-Lüscher et al. 2016). However, these factors gain greater importance only in later phenological phases. It is therefore assumed that the choice of phenology models to simulate early plant development (BBCH09) was appropriate and similar models have also been used in several other studies (Basler 2016; Chuine 2000; Nufer 2013; Fila et al. 2014). To evaluate the different phenology models, RMSE, RMSE′, and NSE were calculated. The RMSE is comparable to results by Fila et al. (2014) who compared different phenology models for grapevine. They computed NSE ranging from 0.26 to 0.46. The NSE for the models used here ranges from 0.62 to 0.92, thus indicating even better fits. When comparing F and CF models (i.e., models solely depending on FU accumulation and models also including CU accumulation), the observations reported by Fila et al. (2014) are confirmed: CF models do not outperform F models under present conditions. However, Fila et al. (2014) concluded that CF models should be preferred when modelling changes in phenology due to climate change since mild winter may influence phenology in a way which cannot be represented by F models. However, Martínez-Lüscher et al. (2016) observed only a small response of BBCH09 in grapevines to changes in chilling temperatures. Hence, the period for predictions used in this study is most likely too short to be influenced by this effect. Additional results from ANOVA show that the choice of phenology model only contributes 4.4% to the total uncertainty, thus putting them far behind the uncertainty associated with model chains, which are responsible for 26.5% of the total uncertainty (data not shown). This is illustrated by the large variability in the change in the time difference between BBCH09 and LDF simulated with ten model chains (Fig. 4). Although the majority of the model chains project a growing difference (i.e., declining frost damage risk), some project the opposite. Interestingly, the model chain projecting the largest decrease in risk (SMHI_RCA_HadCM3Q3) at both sites represents a relatively modest degree of warming compared to the model chain projecting the largest increase at SIO (ETHZ_CLM_HadCM3Q0) (Fuhrer et al. 2014).

In conclusion, the present analysis combining a calibrated phenology model with an ensemble of downscaled climate projections suggests that, in the near future, late spring frost risk in grapevine may either increase or decrease, depending on location and climate model chain. While for the current climate, the risk for frost damage is larger at the warmer site (SIO) compared to that at the cooler site (AIG); for the period 2021–2050, small shifts in both phenology and occurrence of frost (i.e., days with *T*_min_ < 0 °C) lead to a small decrease in frost risk at the warmer site but an increase at the cooler site. However, there are considerable uncertainties related to climate model chains and, consequently, in projected shifts in frost risk. Thus, our results strongly support the use of larger multimodel ensembles when evaluating future climate risks in agriculture.

## Electronic supplementary material

ESM 1 (DOCX 43 kb)
