# Supplementary material for: Changing risk of spring frost damage in grapevines due to climate change? A case study in the Swiss Rhone Valley
Source: Int J Biometeorol. 2018 Jan 24;62(6):991–1002. doi: 10.1007/s00484-018-1501-y (PMC5966476; doi:10.1007/s00484-018-1501-y)
Supplement: Supplementary file 1 — (DOCX 43 kb) [file 484_2018_1501_MOESM1_ESM.docx]

**Supplementary Material**

**Meier M. et al.**

**Phenology models**

Different approaches for simulating grapevine phenology exist in the literature (Chuine, 2000; Nufer, 2013; Fila et al., 2014). The general idea behind these models is that forcing units (FU) must be accumulated up to a certain threshold, depending on the phenological phase of interest. The starting point of this accumulation may be defined in different ways, as explained below. According to Chuine (2000) and Fila et al. (2014), two main model types are widely used to describe grapevine phenology: Forcing (F) models and chilling-and-forcing (CF) models. In the former type, FU with respect to temperature are summed up starting at the same day every year according to a certain date or length of day (i.e. photoperiod). By fitting, and thus fixing this starting day to a specific day of year (DOY), F models propose that dormancy break solely depends on photoperiod and warm temperatures. In the latter type, the starting point regarding FU accumulation is allowed to change from year to year, depending on when a certain threshold for the accumulation of CU is reached. Consequently, CF models depend on winter temperature and can react to mild winters by starting FU accumulation at a later DOY, which implies that breaking dormancy depends on both cold and warm temperatures.

Besides temperature, photoperiod (i.e. length of daylight period) influences plant phenology (Fennell & Hoover, 1991; Ferguson et al., 2011, 2013). Basler (2016) incorporated photoperiod when comparing different phenology models, which he calibrated for six tree species across Europe. He classified the models into three categories: (1) models accounting for ecodormancy (i.e. dormancy release) by calculating FU, including and excluding photoperiod, (2) models accounting for endo- and ecodormancy (i.e. dormancy and dormancy release) by calculating CU and FU, some also adding a response for the photoperiod, and (3) models accounting for the whole dormant period, again, by calculating CU and FU, including temperature responsible for inducing the dormant phase, and including and excluding photoperiod. Here, we applied the first two model types and clustered them according to Basler (2016) into forcing models excluding photoperiod (i.e. F models), forcing models including photoperiod (i.e. F.PP models), chilling-and-forcing models excluding photoperiod (i.e. CF models), and chilling-and-forcing models including photoperiod (i.e. CF.PP models) (see Table S1).

Table S1: Applied clusters of phenology models. The models starting the forcing units (FU) accumulation from a certain day of year (DOY) are the forcing (F) and photoperiod incorporating forcing (F.PP) models. Those models starting FU accumulation after enough chilling units (CU) are summed up, are chilling-and-forcing (CF), and are the chilling-and-forcing (CF) and photoperiod incorporating chilling-and-forcing (CF.PP) models.

|  | **Starting DOY according length of day** | **Starting DOY according summed CU** |
| --- | --- | --- |
| **Temperature** | F models | CF models |
| **Temperature & photoperiod** | F.PP models | CF.PP models |

Due to the limited sample size (i.e. 88 observations), we limited the work to models using six parameters or less. This allowed us to separate the sample into a calibration and a validation part of 63 (i.e. 71.6%) and 25 (i.e. 28.4%) observations, respectively.

**Forcing models**

According to Chuine (2000), two widely used functions in F models are the growing degree-day function (GDD) (eq. S.1, according Murray et al. (1989) and the sigmoid function of mean temperature (SF) (eq. S.2 and eq. S.3), as described by Hänninen (1990) and Kramer (1994):

${FU}_{GDD}=\left\{ \begin{aligned} 0 \mathrm{if} T<T_{bF}, \\ T-T_{bF} \mathrm{if} T\geq T_{bF}. \end{aligned} \right.$ (S.1)

With,

${FU}_{GDD}$: Forcing units according to the growing degree-day function

$T$: Daily mean temperature

$T_{bFU}$: Base temperature below which FU are not accumulated

${FU}_{SF}=\left\{ \begin{aligned} 0 \mathrm{if} T<0, \\ \frac{a}{1+e^{-b\left( T-c \right)}} \mathrm{if} T\geq0. \end{aligned} \right.$ (S.2)

Which can be simplified (see Appendix, chapter 9.2) to

${FU}_{SF,simplified}=\left\{ \begin{aligned} 0 \mathrm{if} T<0, \\ \frac{1}{1+e^{-b\left( T-c \right)}} \mathrm{if} T\geq0. \end{aligned} \right.$ (S.3)

With,

${FU}_{SF}$: Forcing units according to the sigmoid function

$T$: Daily mean temperature

$a,b,c$: Parameters to be fitted by an optimising function, with $a,b,c>0$

Fila et al. (2014) received good results with when calculating FU (and CU) with the beta-type function (Yan and Hunt, 1999; Amaducci et al., 2008) (eq. S.4). To minimize the number of parameters to be optimised, the lower and upper temperature thresholds ($T_{l\beta F}$ and $T_{u\beta F}$, respectively) were fixed, leaving only $T_{o\beta F}$ to be optimised. While $T_{l\beta F}$ was fixed to 0 °C straight away, the value for $T_{u\beta F}$ was searched by conducting different model optimisation runs with $T_{u\beta F}$ being fixed to either 30, 35, 40 or 45 °C. The best results were received when $T_{u\beta F}$ was 35 °C and thus this parameter was fixed by Fila et al. (2014) to that value for further model calibration.

${FU}_{\beta}=\left\{ \begin{aligned} 0 \mathrm{if} T\leq T_{l\beta F} \mathrm{or} T\geq T_{u\beta F}, \\ {\left( \frac{T_{u\beta F}-T}{T_{u\beta F}-T_{o\beta F}} \right)\left( \frac{T-T_{l\beta F}}{T_{o\beta F}-T_{l\beta F}} \right)}^{\left( \frac{T_{o\beta F}-T_{l\beta F}}{T_{u\beta F}-T_{o\beta F}} \right)}\mathrm{if} T_{l\beta F}<T<T_{u\beta F}. \end{aligned} \right.$ (S.4

With,

${FU}_{\beta}$: Forcing units according to the beta-type function

$T$: Daily mean temperature

$T_{l\beta F}$: Lower threshold temperature below which FU are not accumulated, fixed to 0 °C

$T_{u\beta F}$: Upper threshold temperature above which FU are not accumulated, fixed to 35 °C

$T_{o\beta F}$: Optimal temperature leading to the highest FU

**Chilling and forcing models**

While in F models the DOY after which FU are accumulated is fixed, CF models allow for a flexible starting DOY for the FU accumulation. The idea behind is that the plants endodormancy period must end before the growing period starts. This was modelled by incorporating an additional function to add CU. Only after sufficient CU were accumulated (starting from a fixed DOY), FU was accumulated. The functions for the calculation of FU were the same as those used in F models (see above) (Chuine, 2000; Fila et al., 2014). Additionally to these already known FU functions, Chuine (2000) listed two equations to calculate CU, which both may be incorporated in a CF model. The simpler one, with only one parameter to be fitted, is the chilling-days function (CD) (eq. S.5), according Murray et al. (1989). The triangular function (TC) (eq. S.6), as described by Hänninen (1990) and Kramer (1994), is more complex and has three parameters which can be adjusted.

$\boldsymbol{CU}_{\boldsymbol{CD}}\boldsymbol{=}\left\{ \begin{aligned} \boldsymbol{0}\mathbf{if}\boldsymbol{T\geq}\boldsymbol{T}_{\boldsymbol{bC}}\boldsymbol{,} \\ \boldsymbol{1}\mathbf{if}\boldsymbol{T<}\boldsymbol{T}_{\boldsymbol{bC}}\boldsymbol{.} \end{aligned} \right.$ (S.5)

With,

${CU}_{CD}$: Chilling units according to the chilling-days function

$T$: Daily mean temperature

$T_{bC}$: Base temperature, above which CU are not accumulated

$\boldsymbol{CU}_{\boldsymbol{TC}}\boldsymbol{=}\left\{ \begin{aligned} \boldsymbol{0}\mathbf{if}\boldsymbol{T\leq}\boldsymbol{T}_{\boldsymbol{lTC}} \mathbf{or}\boldsymbol{T\geq}\boldsymbol{T}_{\boldsymbol{uTC}}\boldsymbol{,} \\ \frac{\boldsymbol{T-}\boldsymbol{T}_{\boldsymbol{lTC}}}{\boldsymbol{T}_{\boldsymbol{oTC}}\boldsymbol{-}\boldsymbol{T}_{\boldsymbol{lTC}}} \mathbf{if} \boldsymbol{T}_{\boldsymbol{lTC}}\boldsymbol{<T\leq}\boldsymbol{T}_{\boldsymbol{oTC}}\boldsymbol{,} \\ \frac{\boldsymbol{T-}\boldsymbol{T}_{\boldsymbol{uTC}}}{\boldsymbol{T}_{\boldsymbol{oTC}}\boldsymbol{-}\boldsymbol{T}_{\boldsymbol{uTC}}} \mathbf{if} \boldsymbol{T}_{\boldsymbol{oTC}}\boldsymbol{<T<}\boldsymbol{T}_{\boldsymbol{uTC}}\boldsymbol{.} \end{aligned} \right.$ (S.6)

With,

${CU}_{TC}$: Chilling units according to the triangular function

$T$: Daily mean temperature

$T_{lTC}$: Lower threshold temperature, below which CU are not accumulated

$T_{uTC}$: Upper threshold temperature, above which CU are not accumulated

$T_{oTC}$: Optimal temperature, whereas if $T=T_{opt}$, then $CU=1$

Alternatively, Fila et al. (2014) again used the already explained beta-type function to calculate CU (eq. S.7). Unlike when applied for FU, they allowed for all three parameters (i.e. $T_{upr}$, $T_{opt}$ and $T_{lwr}$) to be optimised when modelling CU accumulation.

$\boldsymbol{CU}_{\boldsymbol{\beta C}}\boldsymbol{=}\left\{ \begin{aligned} \boldsymbol{0}\mathbf{if}\boldsymbol{T\leq}\boldsymbol{T}_{\boldsymbol{l\beta C}} \mathbf{or}\boldsymbol{T\geq}\boldsymbol{T}_{\boldsymbol{u\beta C}}\boldsymbol{,} \\ {\left( \frac{\boldsymbol{T}_{\boldsymbol{u\beta C}}\boldsymbol{-T}}{\boldsymbol{T}_{\boldsymbol{u\beta C}}\boldsymbol{-}\boldsymbol{T}_{\boldsymbol{o\beta C}}} \right)\left( \frac{\boldsymbol{T-}\boldsymbol{T}_{\boldsymbol{l\beta C}}}{\boldsymbol{T}_{\boldsymbol{o\beta C}}\boldsymbol{-}\boldsymbol{T}_{\boldsymbol{l\beta C}}} \right)}^{\left( \frac{\boldsymbol{T}_{\boldsymbol{o\beta C}}\boldsymbol{-}\boldsymbol{T}_{\boldsymbol{l\beta C}}}{\boldsymbol{T}_{\boldsymbol{u\beta C}}\boldsymbol{-}\boldsymbol{T}_{\boldsymbol{o\beta C}}} \right)}\mathbf{if} \boldsymbol{T}_{\boldsymbol{l\beta C}}\boldsymbol{<T<}\boldsymbol{T}_{\boldsymbol{u\beta C}}\boldsymbol{.} \end{aligned} \right.$ (S.7)

With

${CU}_{\beta}$: Chilling units according to the beta-type function

$T$: Daily mean temperature

$T_{l\beta C}$: Lower threshold temperature below which CU are not accumulated

$T_{u\beta C}$: Upper threshold temperature above which CU are not accumulated

$T_{o\beta C}$: Optimal temperature leading to the highest CU

**Photoperiod depending forcing models**

In order to account for photoperiod in FU accumulation, F models were adjusted to photoperiod-depending forcing (F.PP) models. Following Basler (2016) and Blümel and Chmielewski (2012), this was done by multiplying the initially calculated FU with an additional, photoperiod-dependent term. While the photoperiod only uses the DOY as input variable and thus can be regarded as fixed, the exponent of this additional term was optimised when fitting the model. This concept was applied to all the three FU accumulating functions (eq. S.8; S.9; S.10):

$\boldsymbol{FU}_{\boldsymbol{GDD.PP}}\boldsymbol{=}\boldsymbol{FU}_{\boldsymbol{GDD}}\boldsymbol{\times}\left( \frac{\boldsymbol{PP}}{\boldsymbol{12}\boldsymbol{h}} \right)^{\boldsymbol{d}}$ (S.8)

With,

${FU}_{GDD.PP}$: Forcing units according to the photoperiod depending growing degree-days function

${FU}_{GDD}$: Forcing units according to the growing degree-days function

$d$: Parameter to be fitted by an optimising function

$PP$: Photoperiod. See eq. 2.22

$\boldsymbol{FU}_{\boldsymbol{SF.PP}}\boldsymbol{=}\boldsymbol{FU}_{\boldsymbol{SF}}\boldsymbol{\times}\left( \frac{\boldsymbol{PP}}{\boldsymbol{12}\boldsymbol{h}} \right)^{\boldsymbol{d}}$ (S.9)

With,

${FU}_{SF.PP}$: Forcing units according to the photoperiod depending sigmoid function

${FU}_{SF}$: Forcing units according to the sigmoid function

$d$: Parameter to be fitted by an optimising function

$PP$: Photoperiod. See eq. 2.22

$\boldsymbol{FU}_{\boldsymbol{\beta.PP}}\boldsymbol{=}\boldsymbol{FU}_{\boldsymbol{\beta}}\boldsymbol{\times}\left( \frac{\boldsymbol{PP}}{\boldsymbol{12}\boldsymbol{h}} \right)^{\boldsymbol{d}}$ (S.10)

With,

${FU}_{\beta.PP}$: Forcing units according to the photoperiod depending beta-type function

${FU}_{\beta}$: Forcing units according to the beta-type function

$d$: Parameter to be fitted by an optimising function

$PP$: Photoperiod. See eq. 2.22

The photoperiod (here defined as the time between sunrise and sunset) was calculated according Fischer et al. (2014) (eq. S.11), with the longitude and latitude of the Swiss Rhone Valley (i.e. 23.45° and 46° N). If the number of days in a year is fixed, as well as the DOY of the vernal equinox, the photoperiod solely depends on DOY for which the photoperiod is calculated. For the Swiss Rhone valley this leads to photoperiods between 8.44 and 15.56 hours and thus the term $\left( \frac{PP}{12h} \right)$ lies between 0.70 and 1.30.

$\boldsymbol{PP=hr.d\times}\frac{\mathbf{cos}^{\boldsymbol{-1}} \left( \boldsymbol{-}\tan\boldsymbol{\Phi} \right)\tan\left( \boldsymbol{tr\times}\sin\left( \frac{\boldsymbol{360^{\circ}}}{\boldsymbol{d.yr}}\left( \boldsymbol{DOY-d.eq} \right) \right) \right)}{\boldsymbol{\pi}}$ (S.11)

With,

$PP$: Photoperiod

$hr.d$: Length of one day. Thus $hr.d=24 hours$

$\Phi$: Latitude. Thus $\Phi=46^{\circ}$ N

$tr$: Tropic. Thus $tr=23.45^{\circ}$ N

$DOY$: Day of year for which the photoperiod is calculated

$d.yr$: Number of days in a year. Set to $d.yr=365 days$

$d.eq$: DOY of the vernal equinox. Set to $d.eq=81 days$

**Photoperiod-depending chilling and forcing models**

This category of photoperiod -depending chilling and forcing (CF.PP) models clusters CF models in which FU accumulation depends on photoperiod. Thus, CU are calculated by the above mentioned CD, TC and beta-type functions, starting from a fixed DOY. As soon as a fitted threshold is reached, FU are accumulated, using the same functions as in the F.PP models above (i.e. photoperiod depending growing degree-days function, sigmoid function and beta-type function).

**Overview of applied and omitted phenology models**

While the starting point for FU accumulation in CF and CF.PP models depends on the timing when the threshold for CU accumulation is reached, it is a parameter to be calibrated for F and F.PP models. To restrain the number of parameters to be fitted, the starting point for CU accumulation was fixed to DOY 244 (i.e. 1 September), following Chuine (2000). Additionally, the thresholds for CU and FU accumulation were calibrated thus adding one parameter to F and F.PP models as well as two parameters to CF and CF.PP models. This led to some models having seven or even eight parameters to be fitted. Due to the limited availability of data, these models were omitted. All phenology models, together with their complexity, are listed in Table S2.

Table S2: Overview of applied and omitted phenology models. All phenology models are listed together with the type of model they belong to (i.e. forcing (F), chilling-and forcing (CF), forcing including photoperiod (F.PP), and chilling-and-forcing including photoperiod (CF.PP) models) as well as the functions they consist of (i.e. forcing units (FU) and chilling units (CU) accumulation) as defined by the corresponding model. Further, the parameters to be fitted and their number is stated, and the model complexity, depending on the number of parameters (i.e. low < 4 parameters ≤ medium < 6 parameters = high). Models with more than 6 parameters were not used.

| **Model** | **Model type and functions** | **Fitted parameters** | **Complexity** |
| --- | --- | --- | --- |
| Growing  degree-day (GDD) | F model  ${FU}_{GDD}$ | 3 parameters:  Starting DOY for FU accumulation ($t_{0}$)  Base temperature for FU accumulation ($T_{bF}$)  FU threshold for BBCH 09 (${Th}_{GDD}$) | low |
| Sigmoid function (SF) | F model  ${FU}_{SF}$ | 4 parameters:  Starting DOY for FU accumulation ($t_{0}$)  Mathematical operands ($b,c$)  FU threshold for BBCH 09 (${Th}_{SF}$) | medium |
| Beta-type function (Beta) | F model  ${FU}_{\beta}$ | 3 parameters:  Starting DOY for FU accumulation ($t_{0}$)  Optimal temperature for FU accumulation ($T_{o\beta F}$)  FU threshold for BBCH 09 (${Th}_{\beta F}$) | low |
| Growing degree-day incl. photoperiod (GDD.PP) | F.PP model  ${FU}_{GDD.PP}$ | 4 parameters:  Starting DOY for FU accumulation ($t_{0}$)  Base temperature for FU accumulation ($T_{bF}$)  Mathematical operand ($d$)  FU threshold for BBCH 09 (${Th}_{GDD.PP}$) | medium |
| Sigmoid function incl. photoperiod (SF.PP) | F.PP model  ${FU}_{SF.PP}$ | 5 parameters:  Starting DOY for FU accumulation ($t_{0}$)  Mathematical operands ($b,c,d$)  FU threshold for BBCH 09 (${Th}_{SF.PP}$) | medium |
| Beta-type function incl. photoperiod (Beta.PP) | F.PP model  ${FU}_{\beta.PP}$ | 3 parameters:  Starting DOY for FU accumulation ($t_{0}$)  Optimal temperature for FU accumulation ($T_{o\beta F}$)  Mathematical operand ($d$)  FU threshold for BBCH 09 (${Th}_{\beta.PP}$) | low |
| Chilling days & growing degree days (CD.GDD) | CF model  ${CU}_{CD}$  ${FU}_{GDD}$ | 4 parameters:  Base temperature for FU accumulation ($T_{bF}$)  Base temperature for CU accumulation ($T_{bC}$)  CU threshold for FU accumulation (${Th}_{CD}$)  FU threshold for BBCH 09 (${Th}_{GDD}$) | medium |
| Chilling days & sigmoid function (CD.SF) | CF model  ${CU}_{CD}$  ${FU}_{SF}$ | 5 parameters:  Base temperature for CU accumulation ($T_{bC}$)  Mathematical operands ($b,c$)  CU threshold for FU accumulation (${Th}_{CD}$)  FU threshold for BBCH 09 (${Th}_{SF}$) | meium |
| Triangular function & growing degree-days (TC.GDD) | CF model  ${CU}_{TC}$  ${FU}_{GDD}$ | 6 parameters:  Lower & upper threshold temperature for CU accum. ($T_{lTC}$ & $T_{uTC}$)  Optimal temperature for CU accumulation ($T_{oTC}$)  Base temperature for FU accumulation ($T_{bF}$)  CU threshold for FU accumulation (${Th}_{TC}$)  FU threshold for BBCH 09 (${Th}_{GDD}$) | High |
| Triangular function & sigmoid function (TC.SF) | CF model  ${CU}_{TC}$  ${FU}_{SF}$ | 7 parameters:  Lower & upper threshold temperature for CU accumulation ($T_{lTC}$ & $T_{uTC}$)  Optimal temperature for CU accumulation ($T_{oTC}$)  Mathematical operands ($b,c$)  CU threshold for FU accumulation (${Th}_{TC}$)  FU threshold for BBCH 09 (${Th}_{SF}$) | high  omitted* |
| Beta-type function & beta-type function (Beta.Beta) | CF model  ${CU}_{\beta}$  ${FU}_{\beta}$ | 6 parameters:  Lower & upper threshold temperatures for CU accum. ($T_{l\beta C}$ & $T_{u\beta C}$)  Optimal temperature for CU accumulation ($T_{o\beta C}$)  Optimal temperature for FU accumulation ($T_{o\beta F}$)  CU threshold for FU accumulation (${Th}_{\beta C}$)  FU threshold for BBCH 09 (${Th}_{\beta F}$) | high |
| Chilling days & growing degree-days incl. photoperiod (CD.GDD.PP) | CF.PP model  ${CU}_{CD}$  ${FU}_{GDD.PP}$ | 5 parameters:  Base temperature for CU accumulation ($T_{bC}$)  Base temperature for FU accumulation ($T_{bF}$)  Mathematical operand ($d$)  CU threshold for FU accumulation (${Th}_{CD}$)  FU threshold for BBCH 09 (${Th}_{GDD.PP}$) | medium |
| Chilling days & sigmoid function incl. photoperiod (CD.SF.PP) | CF.PP model  ${CU}_{CD}$  ${FU}_{SF.PP}$ | 6 parameters:  Base temperature for CU accumulation ($T_{bC}$)  Mathematical operands ($b,c,d$)  CU threshold for FU accumulation (${Th}_{CD}$)  FU threshold for BBCH 09 (${Th}_{SF.PP}$) | high |
| Triangular function & growing degree-days incl. photoperiod (TC.GDD.PP) | CF.PP model  ${CU}_{CD}$  ${FU}_{GDD.PP}$ | 7 parameters  Lower & upper threshold temperature for CU accum. ($T_{lTC}$ & $T_{uTC}$)  Optimal temperature for CU accumulation ($T_{oTC}$)  Base temperature for FU accumulation ($T_{bF}$)  Mathematical operand ($d$)  CU threshold for FU accumulation (${Th}_{TC}$)  FU threshold for BBCH 09 (${Th}_{GDD.PP}$) | high  omitted |
| Triangular function & sigmoid function incl. photoperiod (TC.SF.PP) | CF.PP model  ${CU}_{TC}$  ${FU}_{SF.PP}$ | 8 parameters:  Lower & upper threshold temperature for CU accumulation ($T_{lTC}$ & $T_{uTC}$)  Optimal temperature for CU accumulation ($T_{oTC}$)  Mathematical operands ($b,c,d$)  CU threshold for FU accumulation (${Th}_{TC}$)  FU threshold for BBCH 09 (${Th}_{SF.PP}$) | high  omitted |
| Beta-type function & beta-type function incl. photoperiod (Beta.Beta.PP) | CF.PP model  ${CU}_{\beta}$  ${FU}_{\beta.PP}$ | 7 parameters:  Lower & upper threshold temperatures for CU accumulation ($T_{l\beta C}$ & $T_{u\beta C}$)  Optimal temperature for CU accumulation ($T_{o\beta C}$)  Optimal temperature for FU accumulation ($T_{o\beta F}$)  Mathematical operand ($d$)  CU threshold for FU accumulation (${Th}_{\beta C}$)  FU threshold for BBCH 09 (${Th}_{\beta.PP}$) | high  omitted |

Table S3: Mean and range of fitted parameters in phenology models. The parameters of the different functions making up the phenology models are listed according their number.

| **Parameter** | **Fitted value**  **Mean (Min:Max)** | **N° of parameters** |
| --- | --- | --- |
| Starting day of year (DOY) for forcing unit (FU) accumulation in all forcing (F) and photoperiod incorporating forcing (F.PP) models ($t_{0}$) | 1.78  (-0.06 : 5.94) | 6 |
| Mathematical operand in all photoperiod incorporating (PP) models ($d$) | 3.98  (3.03 : 4.64) | 5 |
| FU to accumulate until budburst (BBCH 09) in growing degree-days (GDD) and growing degree-days incl. photoperiod (GDD.PP) models (${Th}_{GDD}$) | 148.75 FU  (147.71 : 150.26) | 5 |
| Base temperature for FU accumulation in GDD and GDD.PP functions ($T_{bF}$) | 5.55 °C  (5.20 : 5.67) | 5 |
| FU to accumulate until BBCH 09 in sigmoid function (SF) and sigmoid function incl. photoperiod (SF.PP) (${Th}_{SF}$) | 11.00 FU  (8.92 : 12.37) | 4 |
| Mathematical operand in SF and SF.PP functions ($b$) | 0.28  (0.26 : 0.30) | 4 |
| Mathematical operand in SF and SF.PP functions ($c$) | 14.28  (13.37 : 15.67) | 4 |
| FU to accumulate until BBCH 09 in beta-function (Beta) and beta-function incl. photoperiod (Beta.PP) (${Th}_{\beta F}$) | 17.81 FU  (16.77 : 19.15) | 3 |
| Optimal temperature for FU in the Beta function ($T_{o\beta F}$) | 23.14 °C  (22.70 : 23.44) | 3 |
| Chilling units (CU) to accumulate until FU accumulation starts in the chilling days function (CD) (${Th}_{CD}$) | 8.53 CU  (5.33 : 9.93) | 4 |
| Base temperature for CU accumulation in the CD function ($T_{bC}$) | 2.72 °C  (0.65 : 4.76) | 4 |
| CU to accumulate until FU accumulation starts in the triangular function (TC) (${Th}_{TC}$) | 52.43 CU | 1 |
| Lower threshold temperature for CU accumulation in the TC function ($T_{lTC}$) | -4.00 °C | 1 |
| Upper threshold temperature for CU accumulation in the TC function ($T_{uTC}$) | 9.81 °C | 1 |
| Optimal temperature for CU accumulation in the TC function ($T_{oTC}$) | -1.84 °C | 1 |
| CU to accumulate until FU accumulation starts in the beta-function (Beta) (${Th}_{\beta C}$) | 50.71 CU | 1 |
| Lower threshold temperatures for CU accumulation in the Beta function ($T_{l\beta C}$) | -8.31 °C | 1 |
| Upper threshold temperatures for CU accumulation in the Beta function ($T_{u\beta C}$) | 8.69 °C | 1 |
| Optimal temperature for CU accumulation in the Beta function ($T_{o\beta C}$) | 1.12 °C | 1 |

Table S4 shows the results of the validation. The calibrated models led to values of 2.75 to 5.80, 2.50 to 5.57 and 0.92 to 0.62 for RMSE, RMSE’ and NSE, respectively. The ranking of the first six models is the same for all three statistics: (1) SF.PP, (2) CD.SF.PP, (3) Beta.PP, (4) GDD.PP, (5) CD.GDD.PP, and (6) Beta.Beta. While rank seven and eight show differences between the statistics, rank nine to twelve are identical again. These equal results for almost all ranks gave great confidence in the validation. It also becomes obvious, that the PP models, be it F.PP or CF.PP, outperformed models excluding photoperiod.

Table S4: Ranking of phenology models according RMSE, RMSE’ and NSE. Next to the root mean square error (RMSE), the unbiased RMSE (RMSE’) and the Nash-Sutcliffe Efficency index (NSE) are computed with the validation sample for each model. The meaning of the model abbreviations is explained in Table S5.

| **Model** | **RMSE** |  | **Model** | **RMSE'** |  | **Model** | **NSE** |
| --- | --- | --- | --- | --- | --- | --- | --- |
| SF.PP | 2.7495 |  | SF.PP | 2.4929 |  | SF.PP | 0.9153 |
| CD.SF.PP | 3.0984 |  | CD.SF.PP | 2.6533 |  | CD.SF.PP | 0.8924 |
| Beta.PP | 3.1305 |  | Beta.PP | 2.8744 |  | Beta.PP | 0.8902 |
| GDD.PP | 3.7523 |  | GDD.PP | 3.0683 |  | GDD.PP | 0.8422 |
| CD.GDD.PP | 3.8730 |  | CD.GDD.PP | 3.0709 |  | CD.GDD.PP | 0.8319 |
| Beta.Beta | 3.9038 |  | Beta.Beta | 3.5786 |  | Beta.Beta | 0.8292 |
| SF | 4.0988 |  | Beta | 4.0386 |  | SF | 0.8117 |
| Beta | 4.2615 |  | SF | 4.0485 |  | Beta | 0.7965 |
| TC.GDD | 4.3081 |  | TC.GDD | 4.2332 |  | TC.GDD | 0.7920 |
| CD.SF | 4.4721 |  | CD.SF | 4.4261 |  | CD.SF | 0.7758 |
| CD.GDD | 5.5027 |  | CD.GDD | 5.4253 |  | CD.GDD | 0.6606 |
| GDD | 5.7966 |  | GDD | 5.5714 |  | GDD | 0.6234 |

Table S5: Phenology model abbreviations and their meaning. All phenology models accumulate forcing units up to certain threshold. The day this threshold is reached is the modelled day of year for budburst.

| **Model abbreviation** | **Model description according the incorporated functions and its functionality** |
| --- | --- |
| Beta | Starting from a fixed date, forcing units (FU) according the beta-function, solely depending on daily mean temperature, (Beta) are accumulated. |
| GDD | Starting from a fixed date, FU according the growing degree-days function, solely depending on daily mean temperature, (GDD) are accumulated. |
| SF | Starting from a fixed date, FU according the sigmoid function, solely depending on daily mean temperature, (SF) are accumulated. |
| Beta.PP | Starting from a fixed date, FU according the beta-type function incorporating daily mean temperature and daily photoperiod (Beta.PP) are accumulated. |
| GDD.PP | Starting from a fixed date, FU according the growing degree-days function incorporating daily mean temperature and daily photoperiod (GDD.PP) are accumulated. |
| SF.PP | Starting from a fixed date, FU according the sigmoid function incorporating daily mean temperature and daily photoperiod (SF.PP) are accumulated. |
| Beta.Beta | Starting after a threshold for chilling units (CU) accumulation according Beta has been reached, FU according Beta are accumulated, solely depending on daily mean temperature. |
| CD.GDD | Starting after a threshold for CU accumulation according the chilling days function (CD) has been reached, FU according GDD are accumulated, solely depending on daily mean temperature. |
| CD.SF | Starting after a threshold for CU accumulation according CD has been reached, FU according SF are accumulated, solely depending on daily mean temperature. |
| TC.GDD | Starting after a threshold for CU accumulation according the triangular function (TC) has been reached, FU according GDD are accumulated, solely depending on daily mean temperature. |
| CD.GDD.PP | Starting after a threshold for CU accumulation according CD has been reached, FU according GDD.PP are accumulated, depending on daily mean temperature and daily photoperiod. |
| CD.SF.PP | Starting after a threshold for CU accumulation according CD has been reached, FU according SF.PP are accumulated, depending on daily mean temperature and daily photoperiod. |

**References**

Basler D (2016). Evaluating phenological models for the prediction of leaf-out dates in six temperate tree species across central Europe. Agricultural and Forest Meteorology 217: 10-21.

Blümel K, Chmielewski FM (2012) Shortcomings of classical phenological forcing models and a way to overcome them. Agricultural and Forest Meteorology 164: 10–19.

Chuine, I (2000) A unified model for budburst of trees. Journal of Theoretical Biology 207: 337-347.

Fennell A, Hoover E (1991). Photoperiod influences growth, bud dormancy, and cold acclimation in Vitis labruscana and V. riparia. Journal of the American Society for Horticultural Science 116: 270-273.

Ferguson J C, Tarara JM, Mills LJ, Grove GG, Keller M (2011). Dynamic thermal time model of cold hardiness for dormant grapevine buds. Annals of Botany 107: 389-396.

Ferguson JC, Moyer MM, Mills LJ, Hoogenboom G, Keller M (2013). Modeling dormant bud cold hardiness and budbreak in 23 Vitis genotypes reveals variation by region of origin. American Journal of Enology and Viticulture 65: 59-71.

Fila G, Gardiman M, Belvini P, Meggio F, Pitacco A (2014) A comparison of different modelling solutions for studying grapevine phenology under present and future climate scenarios. Agricultural and Forest Meteorology 195: 192-205.

Fischer H, Leuenberger M, Joos F, Stocker T (2014) Introduction to Climate and Environmental Physics. Physics Institute, University of Bern, 129 pp.

Hänninen H (1990) Modelling bud dormancy release in trees from cool and temperate regions. Acta Forestalia Fennica 213: 1-47.

Kramer K (1994) Selecting a model to predict the onset of growth of Fagus sylvatica. Journal of Applied Ecology 31: 172-181.

Murray MB, Cannell MGR, Smith RI (1989) Date of budburst of fifteen tree species in Britain following climatic warming. Journal of Applied Ecology 26: 693-700.

Nufer M (2013) Impact of climate change on grapevine phenology in Switzerland - phenology model for Vitis vinifera cv. Pinot Noir and Chasselas. Master Thesis. Agroscope and ETH, Swiss Federal Institute of Technology Zurich.
